# A step-wise approach towards introduction of an alcohol based hand rub, and implementation of front line ownership- using a, rural, tertiary care hospital in central India as a model

**DOI:** 10.1186/s12913-015-0840-1

**Published:** 2015-04-29

**Authors:** Megha Sharma, Rita Joshi, Harshada Shah, Ragini Macaden, Cecilia Stålsby Lundborg

**Affiliations:** Department of Pharmacology, R. D. Gardi Medical College, Ujjain, MP India; Global Health (IHCAR), Department of Public Health Sciences, Karolinska Institutet, Stockholm, Sweden; Department of Microbiology, R. D. Gardi Medical College, Ujjain, MP India; Infectious Disease Unit, St. John’s Medical College and Research Institute, Bangalore, India

**Keywords:** Hand hygiene, In-house prepared alcohol based hand rub, Confidence building, Finger tip culture-visual portrayal, Front line ownership, Behaviour change, Tertiary care hospital, India

## Abstract

**Background:**

Appropriate hand hygiene is a gold standard to combat healthcare associated infections (HAIs). The World Health Organization (WHO) has recommended alcohol based hand rub (ABHR) as the most effective tool to maintain hand hygiene. In resource poor settings commercially available ABHR is not “economically accessible”. The objectives of this study were to assess the acceptability of, and to build confidence for an in-house prepared (based on WHO guidelines) alcohol based hand rub among healthcare workers (HCWs) using a rural, tertiary care hospital in central India as an example.

**Methods:**

A series of activities were developed and conducted based on the Precede-Proceed model, the Trans Theoretical model of behaviour change, Front line ownership and Social marketing. A modified WHO-ABHR formulation, the ‘test product’ and ‘WHO product evaluation form’ were used for self assessment of acceptability of the ‘test product’. Confidence building activities, as finger tip culture, visual portrayal method and handmade posters, were used in high-risk wards for HAIs, to build confidence for the ‘test product’ in removing transient flora from the hands. A locally developed feedback from was used to evaluate the impact of the activities conducted.

**Results:**

Overall 183 HCWs were enrolled for the assessment of the ‘test product’ (130- doctors and 53 nurses). Out of these 83% (108/130) doctors and 94% (50/53) nurses were satisfied with the ‘test product’. The confidence building activity was conducted with 116 participants (49 doctors). After single use of the ‘test product’, overall a significant reduction was observed for the CFUs on the blood agar plates (0.77 Log^10^, p < 0.001). A complete reduction (100%) in colony forming units on incubated blood agar plates was seen for 13% (15/116) participants. Eighty two percent (95/116) participants expressed their confidence in the ‘test product’.

**Conclusion:**

The self reported acceptance level for the ‘test product’ was high. The use of finger tip culture coupled with the visual portrayal was perceived as a convincing and highly effective way to develop confidence in HCWs. Thus, is the foremost step towards successful introduction of ABHR and can be seen as a model for similar settings.

## Background

Healthcare associated infections (HAIs) are a significant cause for patients’ long term disability, overall increase in morbidity, mortality and financial burden. Incidences of HAIs are reported to be higher in low and middle income countries than in high income countries [1,2]. A major cause for HAIs is hands of healthcare workers (HCWs) [3,4]. Studies have shown that appropriate hand hygiene practice is the most important and cost effective means to prevent and reduce transmission of HAIs [5-7]. The WHO has recognized alcohol based hand rub (ABHR) as a gold standard for hand hygiene for visually or felt clean hands but use of soap and water is recommended for visibly soiled hands.

After the worldwide recognition of effectiveness of the ABHR various commercial, physically attractive and well packed alcohol based products appeared in the markets [8]. The costs of these products are out of reach for many resource poor settings. For such settings, in-house production of cost effective WHO recommended hand rub (HR) formulation is an alternative, without compromising on efficacy [9,10].

Studies particularly from high income countries and a few from low-middle income countries focus on prevention and control of HAIs. Of those even fewer propose any suitable and valid methods to test the level of confidence, tolerability and acceptability of a hand hygiene product under real work conditions [7,11].

According to Precede-Proceed model “An educational diagnosis is needed to design a health promotion intervention, just as a medical diagnosis is needed to design a treatment plan” [12]. According to Prochaska, changing of behaviour is an individual process, thus a campaign is likely to have more impact when it is personalized [13]. A systemic review on the use of behaviour change theory by Edwards et al. also suggests that interventions targeted at experiential thinking and socialization factors designed are more effective [14]. Front Line Ownership (FLO) approach proposes the strength of handing over the ownership of an idea rather than selling the ideas to the workers [15]. The theories and models mentioned above advocates in one way or other to build a relationship with the target or participants over a time.

The need for ABHR was previously explored using focus group discussions among HCWs of the study setting [10]. The main issues that were highlighted were: understaffing, inadequate hand washing (HW) facilities, and lack of clean towels and running water and ABHR in the hospital [10]. In resource limited settings with high patient load the use of ABHR could be preferred for the ease of use, and time consumed compared with conventional hand washing. The use of ABHR may even be more cost effective at times as there is no need for additional staffing, continuous water supply, clean towels, and sinks close to the patient beds. Frequent hand washing with soap and water or use of ethyl alcohol as a hand disinfectant make the skin rough and dry [16]. However, an ABHR contains an emollient which keeps the skin moist and soft. Thus, the introduction of ABHR can be an important step in such settings to promote hand hygiene.

The successful introduction of a cost effective ABHR in order to combat HAIs in a resource poor setting is challenging. The aim of this study was to assess acceptability and tolerance of in-house prepared ABHR and to build capacity and confidence in HCWs from a rural, tertiary care hospital in central India. The long term aim is to facilitate successful hospital-wide introduction of ABHR and to subsequently improve the hand hygiene compliance and effectiveness among HCWs, as per institutional policy.

## Methods

The study was conducted in C. R. Gardi hospital; a rural, tertiary care, teaching hospital (570 beds) affiliated to the R. D. Gardi Medical College. The hospital is situated in a village of Ujjain district, Madhya Pradesh, India. The hospital provides free nursing care and offers other services at low cost to all patients, particularly benefiting the patients from nearby villages, with general poor socio-economic background. In such settings, infection control measures have to be low cost and effective [10].

The social marketing model including four ‘P’s were designated as (i) Product- in house prepared alcohol based hand rub (IH-ABHR), (ii) Price- easy and fast action of the ABHR compared to hand washing, (iii) Place- High risk wards of the facility and (iv) Promotion- the motivation of target participants to use IH-ABHR, was done by using handmade and WHO posters. The cost of ingredients and supply was carried by the funds available within the research project and the management offered managerial and execution support which is also crucial.

**Figure 1:**
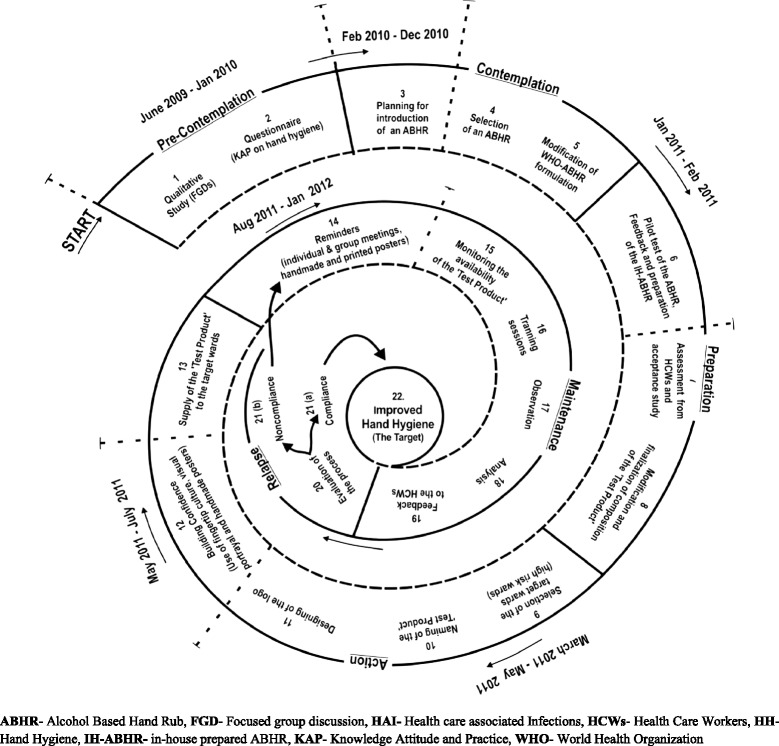
Strategy and time plan to introduce the in-house prepared alcohol based hand rub in a rural, tertiary care teaching hospital in Central India.

To address multiple levels of HCWs in the setting such as, doctors, nurses and auxiliaries, a step wise approach was developed for the targeted intervention by integrating theories such as the Precede-Proceed, Trans Theoretical model of behaviour change, FLO and Social marketing models [12,13,15]. The WHO slogan “Clean hands- Save lives” was used to support the introduction of in-house prepared ABHR [9]. The time plan of the strategy and the complete design of the strategy developed for the introduction of an ABHR in the hospital and evaluation of the process; based on Trans Theoretical model is shown in Figure 1 [13].

### Ethical approval

The project was approved by the Ethics committee of R. D. Gardi Medical College, Ujjain, India: 169/2011.

### Step I: Selection, modification, testing and assessment

#### Selection, modification and testing of the IH-ABHR

WHO-formulated ABHR was selected because of its cost effectiveness, high level of efficacy and possibility of in-house preparation [9]. All the ingredients were obtained from local; International organization for standardization (ISO) certified companies. Appropriate modifications were done in WHO formulation as per the strengths of locally available ingredients to produce IH-ABHR. The product was tested for its effectiveness in the laboratory by using the glove juice method [17].

#### Assessment of the IH-ABHR by the participants for acceptance and tolerability

Nine sessions (25 to 45 minutes) were organized separately for doctors (6 sessions) and nurses (3 sessions) in March-May 2011. The skin of the hands of each participant was checked for cuts, wounds or evidence of any allergic reaction, prior to proceeding for the individual assessment of the IH-ABHR. All sessions were conducted under working practice conditions to assess the tolerance and acceptability of the IH-ABHR. The team then proceeded to demonstrate the application of ABHR on to the hands in accordance with the WHO technique for correct hand coverage [18]. Subsequently, the HCWs were instructed to withdraw 3–5 ml of the IH-ABHR into their cupped palms and to spread it all over the hands in the manner demonstrated. A modified version of ‘WHO product evaluation form’ was used as assessment form for subjective evaluation immediately after single use of the IH-ABHR. The form was fully structured and all the parameters were recorded on a 7 point scale (Likert like scale) to obtain an overall acceptability score [9]. The most unpleasant experience, for example, for smell and colour could be 1 and the most pleasant experience would be rated as 7 on the scale. The assessment was done through individual anonymous reporting by the HCWs.

### Step II: Final modification, selection of target wards and front line ownership

#### Final modification of the IH-ABHR

Based on the assessment by the participants in Step I, the formulation of the IH-ABHR was modified. The modified product was referred to as the ‘test product’. The effectiveness of the ‘test product’ was tested by the glove juice method [17].

#### Selection of the target wards

The wards at higher risk of spread of HAIs were selected for the initial introduction of the ‘test product’ such as medical intensive care unit (ICU), neonatal ICU, obstetrics and gynaecology, surgery and surgical ICU.

#### Front line ownership

The final modified ‘test product’ from Step I, was named by the project team, using a combination of words from English and Hindi languages. A logo was designed by MS and RJ, approved by all authors and produced as a sticker for the product. The ‘test product’ was filled in the dropping bottles labelled with specifically designed logo. The product was used for next steps as indicated in Figure 1.

### Step III: Building confidence

After assessment of ‘acceptance and tolerability’ by the participants, the next step was to build confidence for the ‘test product’. This part of the strategy was conducted in three stages from May to July 2011 at the high risk wards. Eleven unannounced visits were made between 9 am to 12 noon, each ward was visited 2–3 times. The HCWs who were present during the ward visits and gave verbal consent for participation were included once in the study.

#### Fingertip culture and visual portrayal

The techniques used for the activity were standardized and piloted in the microbiology laboratory before respective studies. The composition and importance of the use of ABHR was explained to the participants in every visit. Standard methods were followed to take fingertip impressions of working hand of HCWs on blood agar plates before and after use of the ‘test product’ [19].

The inoculated blood agar plates were incubated at 37°C for 18–24 hours. Microorganisms were identified by using standard laboratory techniques [19]. The types and number of colony forming units (CFUs) of bacteria were noted before and after use of the modified ‘test product’ , and percentages of reduction in CFUs and Log^10^ were calculated for each individual. To maintain confidentiality, the incubated blood agar plates were shown to the HCWs, individually.

#### Handmade posters

After getting the fingertip impression on blood agar plates an attempt was made to improve hand hygiene by conveying messages through handmade posters. Eight posters with different messages were prepared before the ward visits using drawing sheets. The posters contained ‘gain framed messages’ , written in English or Hindi. The palms of the participants were painted with bright poster colours and hand prints were given on these posters. At the end of the activity, soap and water was provided to clean the colour from the hands and the method of air drying was demonstrated to the participants. The purpose was to convey the message that hand washing with soap and water is recommended for visibly soiled hands.

#### Response to the confidence building study

A structured feedback form was developed to assess the level of confidence among the HCWs for the ‘test product’ , and to explore the success rate of the combination of activities developed and conducted for the purpose. After having been shown the finger-tip cultured incubated blood agar plates, all 116, participants of the fingertip culture and visual portrayal activity filled the form anonymously.

### Step IV: Supply

The labelled bottles with the ‘test product’ were placed at most appropriate places in the high risk target wards such as; in dressing trays and a few were mounted on walls of wards.

### Step V: Reminders

The handmade posters and the “My 5 moments of hand hygiene” posters, available on WHO web site, were printed, framed and mounted in the facility and regular individual and group meetings were conducted in the target wards to serve as reminders to the HCWs [4].

### Data management and analysis

The data was entered in EPI Info (version 3.1) and Excel soft ware and was analyzed using EPI Data analysis and Stata 10.0 (Stata Corp College Station, Texas, USA) software. T-tests were performed for one mean hypothesis test, with null hypothesis. P values < 0.001 were considered to be significant for all except for *Micrococci* (p < 0.01).

## Results

### Step I: Selection, modification, testing and assessment

#### Assessment of the IH-ABHR by the participants, for acceptance and tolerability

In total, 183 HCWs (130 doctors and 53 nurses) assessed the IH-ABHR for acceptance and tolerability. Of 130 doctors, majority (96/130, 74%) were males while most of the nurses were females (45/53, 85%, p < 0.001). Sixty five percent of doctors (85/130) and 74% nurses (39/53) graded the colour of the product as ‘pleasant’ , while 48% (63/130) and 26% (14/53) nurses graded the smell as ‘unpleasant’. Forty two percent (54/130) doctors and all nurses (53/53, p < 0.001) reported that they were (sometimes) using pure ethyl alcohol for hand hygiene in the hospital. Twenty four percent doctors (31/130, p < 0.0001) doctors were using self purchased commercial hand rubs (Table 1: Part A and Part B).

**Table 1:** **Responses for the assessment of the 'test product’, after single use of the in-house prepared alcohol based hand rub (using a feedback form on a 7 point Likert like scale) in a rural tertiary care hospital in Central India**

**Part A**	**Doctors n = 130 (100%)**				**Nurses n = 53 (100%)**			
	**Male-96, Female-34**				**Male-8, Female-35**			
**Questions**	**Details of the codes**			**No reply**	**Details of the codes**			**No reply**
1. ‘What is your opinion of the test product’ for hand hygiene?*	**Unpleasant**		**Pleasant**		**Unpleasant**		**Pleasant**	
**a. **Colour	21 (16)	18 (14)	85 (65)	6 (5)	5 (10)	9 (17)	39 (74)	
**b. **Smell	63 (48)	13 (10)	54 (42)	-	14 (26)	5 (9)	34 (64)	-
**c.** Application	24 (18)	13 (10)	93 (72)	-	4 (8)	2 (4)	47 (89)	-
**d. **Texture	**Sticky**		**Not sticky**		**Sticky**		**Not sticky**	-
	17 (13)	14 (11)	99 (76)	-	5 (9)	6 (11)	42 (79)	
**e. **Irritation	**Irritating**		**Not irritating**		**Irritating**		**Not irritating**	-
	15 (12)	9 (7)	105 (81)	1 (1)	**_**	4 (8)	49 (92)	
**f.** Drying effect	**Much**		**None**		**Much**		**None**	-
	52 (40)	24 (18)	53 (41)	1 (1)	33 (62)	7 (13)	13 (25)	
**g. **Ease of use	**Difficult**		**Easy**		**Difficult**		**Easy**	-
	11 (8)	5 (4)	112 (86)	2 (2)	1 (1)	1 (1)	51 (96)	
**h. **Speed of drying	**Slow**		**Fast**		**Slow**		**Fast**	-
	19 (15)	9 (7)	101 (78)	1 (1)	13 (24)	10 (19)	30 (57)	
**i.**Overall evaluation	**Dissatisfied**		**Satisfied**	-	**Dissatisfied**		**Satisfied**	-
	17 (13)	15 (12)	98 (75)		4 (8)	2 (4)	47 (89)	
**2. Rate the differences between the ‘test product’ and the product used by you in the hospital?**	**Major**		**Minor**		**Major**		**Minor**	-
	65 (50).	14 (11)	33 (25)	18 (14)	51 (96)	1 (2)	1 (2)	
**3. Which product do you prefer?**	**Usual product**		**'Test product'**		**Usual product**		**'Test product'**	
	41 (32)	32 (25)	55 (42)	2 (2)	4 (8)	2 (4)	46 (87)	1(2)
**Evaluation of the 'Test Product'**	**Abnormal**		**Normal**		**Abnormal**		**Normal**	
**a.** Appearance (supple, red, blotchy, rash)	12 (9)	4 (3)	113 (87)	1 (1)	1 (2)	0	52 (98)	-
**b.** Intactness (abrasion, fissures)	15 (12)	2 (2)	112 (86)	1 (1)	2 (4)	0	51 (96)	-
**c. **Moisture content (dryness	18 (14)	9 (7)	102 (78)	1 (1)	4 (8)	0	49 (92)	-
**d. **Sensation (itching, burning, soreness)	17 (13)	4 (3)	108 (83)	1 (1)	3 (6)	1 (2)	49 (92)	-
**4. How would you assess the overall integrity of the skin on your hands?**	Altered		Perfect		Altered		Perfect	-
	14 (11)	10 (8)	101 (78)	5 (4)	1 (2)	2 (4)	50 (94)	
**Part B**								
5a. Are you using any hand rub during hospital work?**	**Yes**		**No**		**Yes**		**No**	-
	70 (54)	-	57 (44)	3 (2)	53 (100)	-	0	
5b. What type of Hand rub are you using presently?**	**Own**		**Spirit**		**Own**		**Spirit**	-
	31 (24)	-	54 (42)	45 (35)	-	-	53 (100)	
6. Do you think that the ‘Test product’ could improve your hand hygiene compliance? **	**Yes**		**No**		**Yes**		**No**	
	90 (69)	9 (7)	28 (22)	3 (2)	49 (93)	-	3 (6)	1
7. Would you like the 'Test product' to be supplied in the hospital? **	**Yes**		**No**		**Yes**			**-**
	119 (92)	-	7 (5)	4 (3)	52 (98)	-	1 (2)	

**For easy tabular presentation we have indicated No = 7 for questions; 5a,6 and 7 in the table, Own: own purchased commercially available ABHR.

Decimals are rounded off to the nearest number.

Ninety doctors out of 130 (69%) and 49 nurses of 53 (93%) felt that their compliance with hand hygiene would improve by using the IH-ABHR. Majority of the doctors (119/130; 92%) and almost all (52/53; 98%) nurses recommended to supply the IH-ABHR in the hospital.

### Step II: Modification and front line ownership

#### Final modification of the IH-ABHR

Based on the assessment by the HCWs, the IH-ABHR was modified by reducing the quantity of water by 14 ml, increasing the quantity of glycerol by 1 ml, adding 4 ml of rose extract and increasing the quantity of ethyl alcohol by 9 ml to get the ‘test product’. The final composition of the modified ‘test product’ is presented in Table 2.

**Table 2:** **Comparison of the compositions of WHO-ABHR formula and the formula modified in a rural tertiary care hospital in Central India to prepare the ‘Micro** ***Kavach*** **’**

**Components**	**WHO- ABHR formula**	**Formula of the ‘Micro** ***Kavach*** **’**
	**(80% alcohol/liter)**	**(80% alcohol/liter)**
Ethyl alcohol	96%: 833.3 ml	95%: 842 ml
Hydrogen peroxide	3%: 41.7 ml	3%: 41.7 ml
Glycerol	98%: 14.5 ml	98%: 15.5 ml
Water	110.8 ml	96.8 ml
Rose extract	NIL	4 ml
Total Amount	1000 ml	1000 ml

ABHR- Alcohol based hand rub, WHO-World Health Organization.

#### Naming the ‘test product’ and designing a logo

The modified ‘test product’ was named as ‘Micro*-Kavach*’ , where “Micro” refers to “micro organisms” and “*Kavach*” (Hindi) which means “to protect”, with the meaning to protect both patients and HCWs from the micro-organisms.

**Figure 2:**
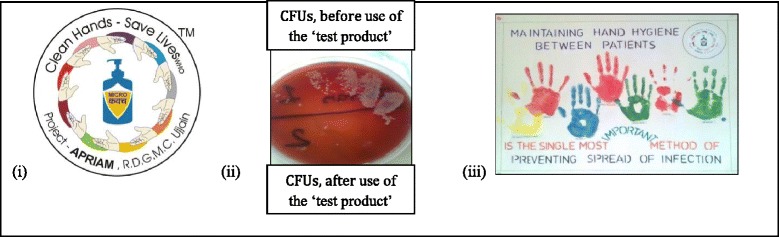
Building confidence- **(i)** The logo **(ii)** Blood agar plate displaying colony forming units (CFUs), **(iii)** Handmade poster.

The logo was designed for the ‘test product’; in consultation with a member of the ‘Clean care-safer care’ team at WHO, Geneva. The logo contains the WHO slogan: “Clean Hands- Save Lives” and products’ name- ‘Micro *Kavach*’ , Figure 2:1. The name i.e. ‘Micro*-Kavach*’ and the logo has now become a registered trademark in India.

### Step III: Building confidence

#### Fingertip culture and visual portrayal

A total of 116 participants (49 doctors and 67 nurses) were included with 116 BA plates in this step (Table 3). Of all participants 63% (31/49) doctors and 16% (11/67, p < 0.001) nurses were males. Overall four different types of bacteria, including potentially pathogenic bacteria, were seen on the blood agar plates before use of ‘Micro *Kavach*’. Figure 2:2 shows one of the incubated blood agar plates presenting the microbial growth before and after use of the ‘Micro *Kavach*’.

**Table 3:** **Microorganisms found on blood agar plates of 116 participants before and after use of 'test product' in Step III- 'Building confidence' in a rural tertiary care hospital in Central India**

**Type of microorganism on blood agar plates (116)**	**Type of microorganisms found on number of blood agar plates n(%)**	**CFU count beforeuse of IH-ABHR**	**CFU count after single use of IH-ABHR**	**CFU count (Log** ^**10**^ **) before use of IH-ABHR**	**CFU count (Log** ^**10**^ **) after single use of IH-ABHR**	**Log** ^**10**^ **difference in reduction of CFUs**
*CONS*	85 (73)	2799	352	3.45	2.547	0.903*
*Staphylococcus aureus*	39 (34)	1927	288	3.29	2.46	1.92*
*Bacillus subtallis*	95 (82)	580	277	2.8	2.44	0.77*
*Micrococci*	23 (20)	285	31	2.46	1.49	0.95^#^
Total	-	5592	948	3.748	2.977	0.771*

CONS-*Coagulase Negative Staphylococci*, *p value <0.001, ^#^p value < 0.01.

*Coagulase Negative Staphylococci* (CONS) were seen in 85/116 (73%) blood agar plates, *Staphylococcus aureus* (*S. aureus*) in 39 (34%), *Bacillus subtilis* in 95 (82%), *Micrococci* in 23 (20%) and gram negative *bacilli* in 17/116 blood agar plates (15%). Majority (101of 116, 87%) of blood agar plates showed two or more types of bacteria (Table 3). After single use of the ‘Micro *Kavach*’ , overall a significant reduction was observed for the CFUs on the blood agar plates (0.77 Log^10^, p < 0.001). The reduction was statistically significant also for individual organisms (Table 3). Majority of blood agar plates presented 75-99% reduction in CFUs (61/116; 53%), and 15 plates (13%) showed 100% reduction. The CFU reduction was less than 50% in 14 blood agar plates.

#### Handmade posters

The handmade posters were framed and displayed in front of the nursing stations of respective wards. After taking the consent, name of each participant was displayed below respective hand prints. Figure 2:3 displays one of such handmade posters.

#### Response to the 'Building confidence' study

All the 116 participants were given a form. After seeing the incubated blood agar plates, 82% (95/116) participants stated that they were highly convinced about the importance and the effectiveness of the ‘Micro *Kavach*’ for maintaining hand hygiene. Almost all participants felt that the fingertip culture and visual portrayal method improved their knowledge and confidence for 'Micro *Kavach*' to maintain hand hygiene. Most (90%) of the HCWs found the whole exercise enjoyable, interesting and effective.

#### Step IV: Supply

The ‘Micro *Kavach*’ was supplied in the targeted wards viz, medical intensive care unit, neonatal intensive care unit, obstetrics and gynecology, surgery and surgical intensive care unit. Due to high and repeated demand of the ‘Micro *Kavach*’ from other wards, the supply was extended to other wards such as pulmonary medicine and orthopedics and a few out-patient departments of the hospital since January 2012.

### Step V: Reminders

The handmade and commercially prepared posters were mounted on the walls in the hospital and were easily visible to people passing by. Several departmental meetings were conducted to discuss the importance of hand hygiene. The team made visits to the target wards to reiterate the importance of the maintaining hand hygiene through ‘Micro *Kavach*’ and where it can be procured from.

## Discussion

The importance of hand hygiene in prevention of transmission of infection was recognized in the 19^th^ century and convincing the HCWs for proper hand hygiene compliance is a challenge since then [20,21]. Skin dryness, irritation, lack of time, work load, under staffing, forgetfulness, poor access to sinks, inadequate knowledge of hand hygiene recommendations and unavailability of ABHR and so on, are consistent contributors for poor compliance of hand hygiene since then [7,18,22,23]. Financial constraints in a healthcare setting hinder to build infrastructural facilities or provide materials necessary for hand washing such as; sinks, running water pipelines, soap, clean towels and the possibility of a regular supply of commercially prepared ABHRs [10].

Providing a low cost ABHR is thus an effective alternative for such settings [9,10]. The use of hand rub is preferred over hand wash for hands not visibly soiled. The use of hand rub is less irritating, requires less time and it could be made easily available [16,24,25].

Non-compliance for maintaining hand hygiene have been reported equally from the settings in high and low-middle income countries [7,21,25]. Thus, the availability of an ABHR has to be coupled with motivation and compliance among the HCWs in order to make the intervention a success. Improving hand hygiene is related to behaviour change of an individual. Hence, non involvement of the HCWs in designing and development of the strategy and poor relationships with the co-workers and undefined policy to address the multiple levels of HCWs are main reasons for the failures of such campaigns [20,26,27]. In the present study, we have addressed the importance of involvement of HCWs throughout the process of formulation and introduction of in-house prepared ABHR in the setting. Various levels of HCWs including administrators, doctors, and nurses were strategically addressed during the process; based on the Precede-Proceed model.

In this study, self assessments regarding skin intactness, moisture content and sensation of the product were reported to be ‘satisfactory’ by a high percentage of the participants. The moisture content in the IH-ABHR was appreciated by a higher number of nurses than the doctors. This could be explained as all the nurses had previously been using 95% ethyl alcohol without any emollient. This might further motivate the HCWs of the setting to use the ‘Micro *Kavach*’. Studies say that alcohols such as ethyl alcohol are often less damaging than detergents but they can also cause dryness and skin irritation [28,29].

The ethyl alcohol; commonly referred to locally as spirit, is supplied by the hospital management to maintain aseptic conditions during routine clinical work. In our study, 42% of doctors and all nurses reported to use spirit to disinfect their hands. The practice might be present due to unavailability of an ABHR in the hospital [11,28]. The use of spirit causes dryness of skin due to absence of any emollient in it whereas an emollient is present in an ideal ABHR which makes the skin softer.

A comparatively higher percentages of doctors (78%) compared to the nurses (57%) felt that the speed of drying of the IH-ABHR was ‘very fast’. This response was probably due to the fact that all the nurses had a practice of using ethyl alcohol which dries faster than any ABHR. The IH-ABHR contained an emollient (glycerine) and a higher amount of water compared to the ‘spirit’ which extends time of drying. Despite this, collectively 76% of HCWs, and a higher percentage of nurses (69% of doctors and 93% of nurses), expressed the view that their hand hygiene compliance will be improved by the use of the IH-ABHR. Overall 71% participants stated to prefer the IH-ABHR over their usual HR products (doctors: 42% and nurses: 87%, p < 0.01). This might be because of the skin softness felt by nurses after the use of IH-ABHR. Thus overall, high satisfaction was achieved for the IH-ABHR in both categories of HCWs.

Involvement of HCWs was ensured in every step of the strategy to make the HCWs feel as the 'decision maker' and the 'sellers' of the in-house prepared ABHR and the message 'maintain hand hygiene'. For example preparation and display of the handmade posters and the final formulation of the ‘Micro *Kavach*’; based on the assessment and feedback by the participants. The anonymous structured assessment forms probably allowed the participants to record their views in an open and unbiased manner. Of the doctors, 48% scored the smell of the IH-ABHR as ‘unpleasant’. Many of these doctors were using an own purchased commercially prepared hand rub that usually contains varied and strong fragrances. This indicated the need to add a fragrance to the IH-ABHR. The addition of rose extract and the larger proportion of the emollient to the formulation might have positively influenced the acceptability and confidence rate towards the final ‘test product’ among the HCWs.

In the same context, the designing of the logo for the in-house prepared ABHR and the name ‘Micro *Kavach*’ was done. Use of a word from the local language, and involvement in the process to modify the formulation of the IH-ABHR might have inculcated a sense of ownership together with a responsibility for maintaining hand hygiene among the participants.

Confidence building is the most important step to convince HCWs for the effectiveness of a new product in a setting, and this confidence can be gained through demonstrating products' efficacy. Researchers have used various methods to show the efficacy of ABHR in settings [27,29,30]. Kampf G et al. supplemented the use of ABHR by fluorescent dye and the hands were assessed under UV torch light to show the presence and reduction of bacterial colonization [31]. Our aim was to design and implement low cost methods to build confidence towards the ‘Micro *Kavach*’; thus purchase of fluorescent dye and UV torch was not considered.

Plante-Jenkins et al. demonstrated the effectiveness of ABHRs in reducing bacteria present on the hands of HCWs by an exercise- “ABHR challenge” with 109 HCWs and thereby to encourage appropriate hand hygiene. The decrease in the fingertip bacterial colony counts (from 28%-100%) after using the hand rub; helped them to convey the message [32]. In our study both visual and percentage reduction in CFU count were illustrated to 116 HCWs by showing the incubated blood agar plates. This method succeeded in creating confidence in the efficacy of ‘Micro *Kavach*’ within the participants. Most of the HCWs rated ‘Micro *Kavach*’ as a convenient and effective way to reduce bacteria from their hands. Less than 50% reduction in CFU count was observed in a few blood agar plates. This might be due to for example, the use of improper hand rub technique indicating at the need for training [33,34]. The fingertip culture method was however, used as a method to convince the users towards the effectiveness of the product, and not as a method of testing the antimicrobial efficacy of the ‘test product’.

To improve the hand hygiene compliance in clinical practice, behavioral change is needed, which is a complex and long term process. Studies related to behavioral changes need to be framed in a contextually appropriate and accepted way. Few studies conducted, all in high income countries, have shown a beneficial effect of use of educational and motivational strategies to increase the rate of use of hand hygiene products [35,36].

Here, easy, interesting and non threatening activities were used to convey the message in an effective and acceptable manner. These activities also helped HCWs to remember and retain the message for a longer period and helped in better compliance. However, to study the degree of compliance, its sustainability and assessment of any dermatological side effects for introduced IH-ABHR is the next step of the process.

Many studies have suggested using professionally produced, glossy, colourful posters for selling hand hygiene messages [34,35], we found that the handmade posters with hand prints of individual HCWs and their name on it gave them a feeling of being a part of the endeavour and thus “ownership of the product and process (FLO)”. This in turn encourages the acceptance of the ‘Micro *Kavach*’ [13,15].

The acceptance and tolerance level among HCWs was promising and encouraged the team to move to the next step i.e. to introduce the ‘Micro *Kavach*’ in the hospital.

### Methodological considerations

The activities included an average of 74% (77% of total doctors and 71% of nurses) of total HCWs enrolled in the hospital. Results of a single time point measure in one setting could obviously not be generalized to other settings as it is. Yet, in view of the high acceptance and demand for the ‘test product’ in the setting, similar results can be anticipated from elsewhere. Thus, we recommend to use the process as a model by relevantly modifying the tools to build confidence for in-house prepared ABHR in similar settings. The emphasis of this paper has been on adaption and introduction of the IH-ABHR and not on change in behavior.

## Conclusion

Acceptability is of great importance for the introduction of a new product for maintaining hand hygiene in a setting. Our results show that the ‘test product’ was well tolerated and accepted by most of the participants. The results have highlighted the high impact of use of personalized methods and FLO design of the study. The use of fingertip culture method coupled with the visual portrayal and handmade poster was a simple, convincing, relatively inexpensive, enjoyable and self motivational approach to develop confidence and to achieve the acceptability of in-house prepared ABHR. Despite the use of relatively simple dispensing bottles, a colourless preparation (unlike many commercial products), ‘Micro *Kavach*’ was well accepted in the setting with a high demand for its supply in the wards. Dedicated and enthusiastic infection control teams that are seeking a way to facilitate the introduction of ABHR can apply this low cost model in their settings with appropriate modification.

## Abbreviations

ABHR: Alcohol based hand rub
CFU: Colony forming unit
CONS: *Coagulase Negative Staphylococci*
C. R. Gardi hospital: Chandrikaben Rashmikant Gradi hospital
FLO: Front line ownership
HAI: Healthcare associated Infection
HCW: Health care worker
HH: Hand hygiene
HR: Hand rub
HW: Hand washing
IH-ABHR: In-house prepared alcohol based hand rub
ISO: International organization for standardization
R. D. Gardi Medical College: Ruxmaniben Deepchand Gardi Medical College
UV: Ultra violet
WHO: World Health Organization
